# Supplementary material for: Exploring the Influence of Growth-Associated Host Genetics on the Initial Gut Microbiota in Horses
Source: Genes (Basel). 2023 Jun 27;14(7):1354. doi: 10.3390/genes14071354 (PMC10379381; doi:10.3390/genes14071354)
Supplement: Supplementary file 1 [file genes-14-01354-s001.zip › Figure S1.pdf]

Figure S1. The relative abundances of gut microbiota according to SNP genotypes in remaining 28 associations

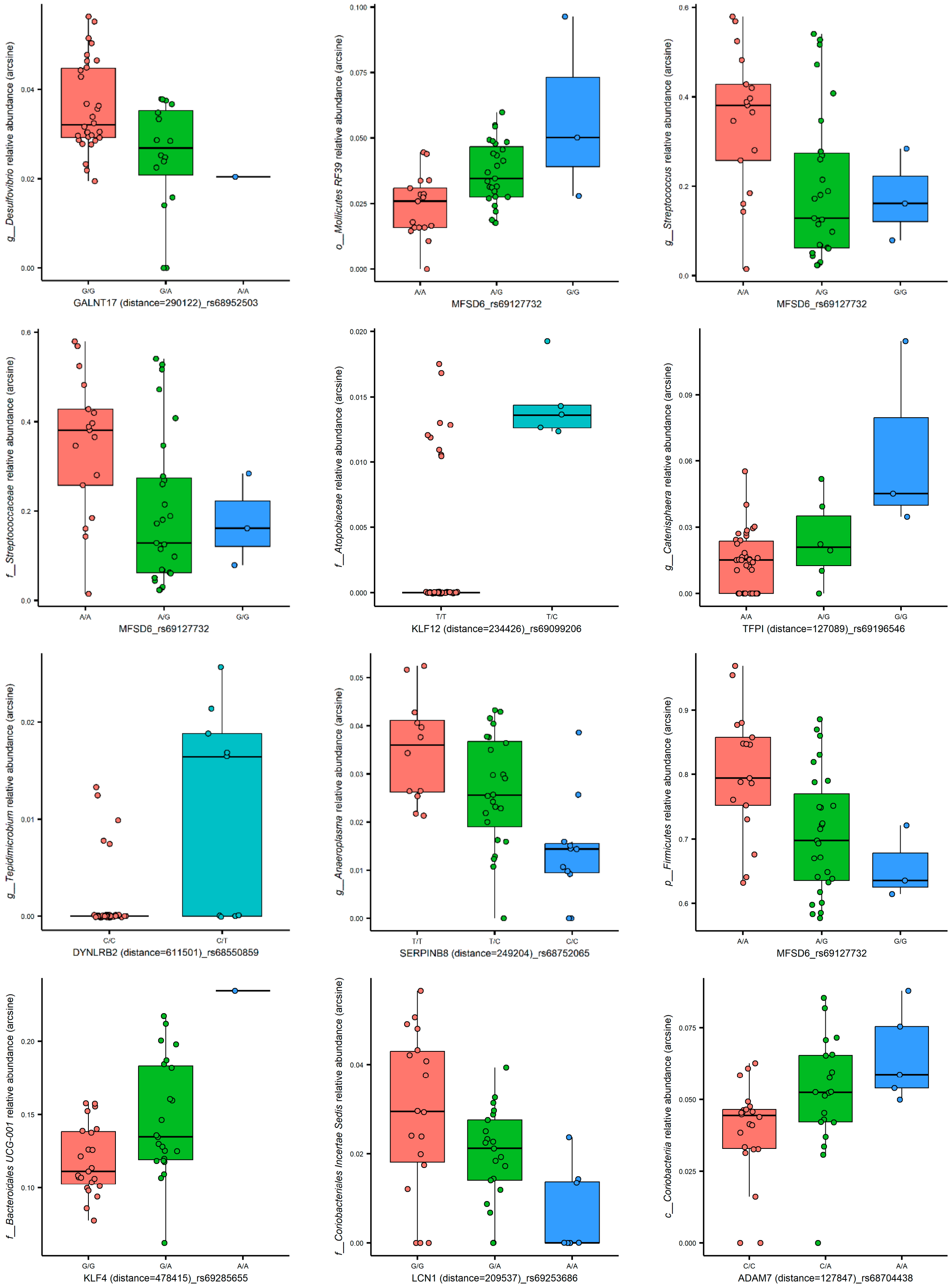

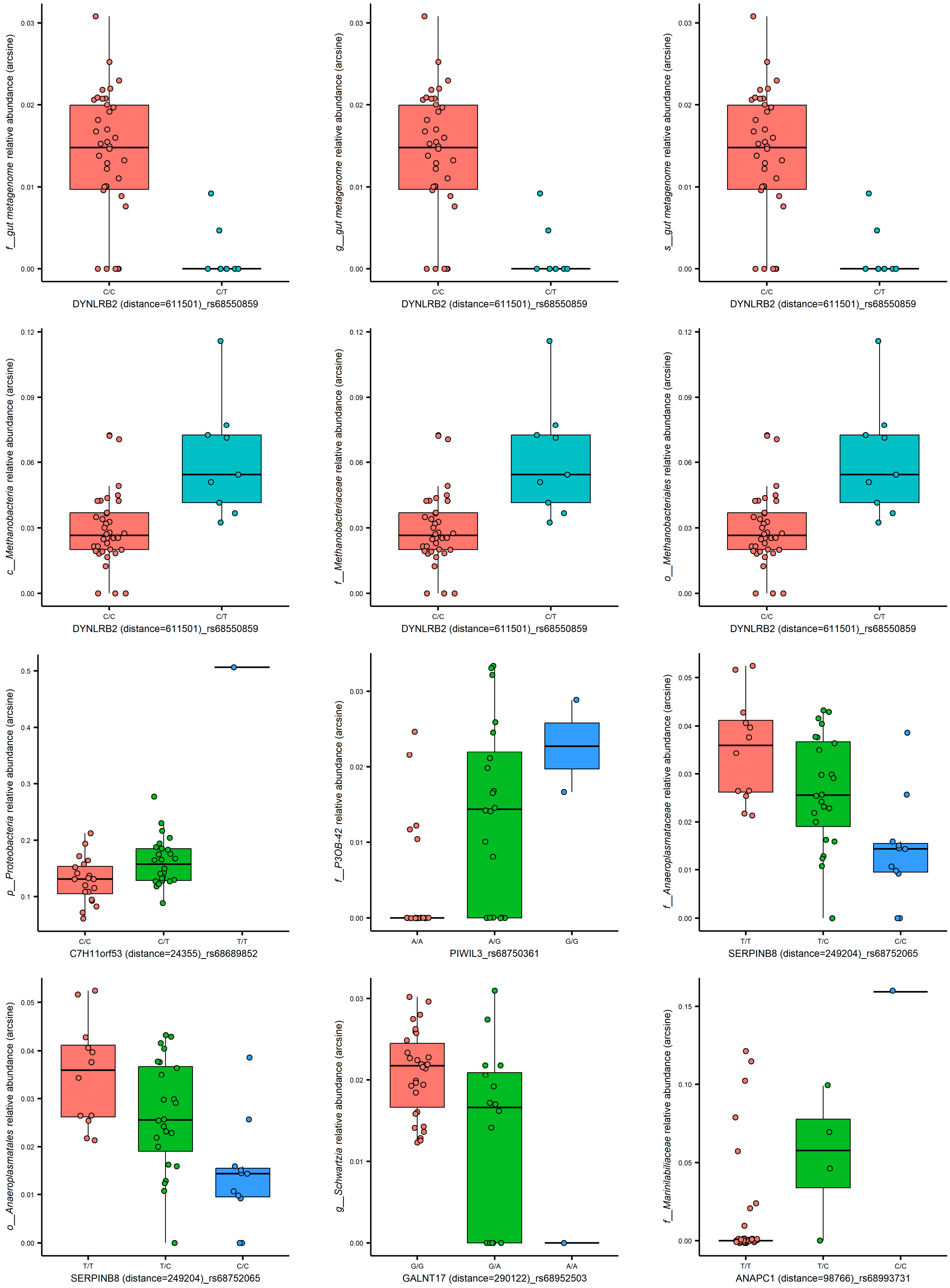
f__gut metagenome relative abundance (arcsine)
DYNLRB2 (distance=611501)_rs68550859
g__gut metagenome relative abundance (arcsine)
DYNLRB2 (distance=611501)_rs68550859
s__gut metagenome relative abundance (arcsine)
DYNLRB2 (distance=611501)_rs68550859
c__Methanobacteria relative abundance (arcsine)
DYNLRB2 (distance=611501)_rs68550859
f__Methanobacteriaceae relative abundance (arcsine)
DYNLRB2 (distance=611501)_rs68550859
o__Methanobacteriales relative abundance (arcsine)
DYNLRB2 (distance=611501)_rs68550859
p__Proteobacteria relative abundance (arcsine)
C7H11orf53 (distance=24355)_rs68689852
f__P3OB-42 relative abundance (arcsine)
PIWIL3_rs68750361
f__Anaeroplasmataceae relative abundance (arcsine)
SERPINB8 (distance=249204)_rs68752065
o__Anaeroplasmatales relative abundance (arcsine)
SERPINB8 (distance=249204)_rs68752065
g__Schwartzia relative abundance (arcsine)
GALNT17 (distance=290122)_rs68952503
f__Marinilabiliaceae relative abundance (arcsine)
ANAPC1 (distance=98766)_rs68993731

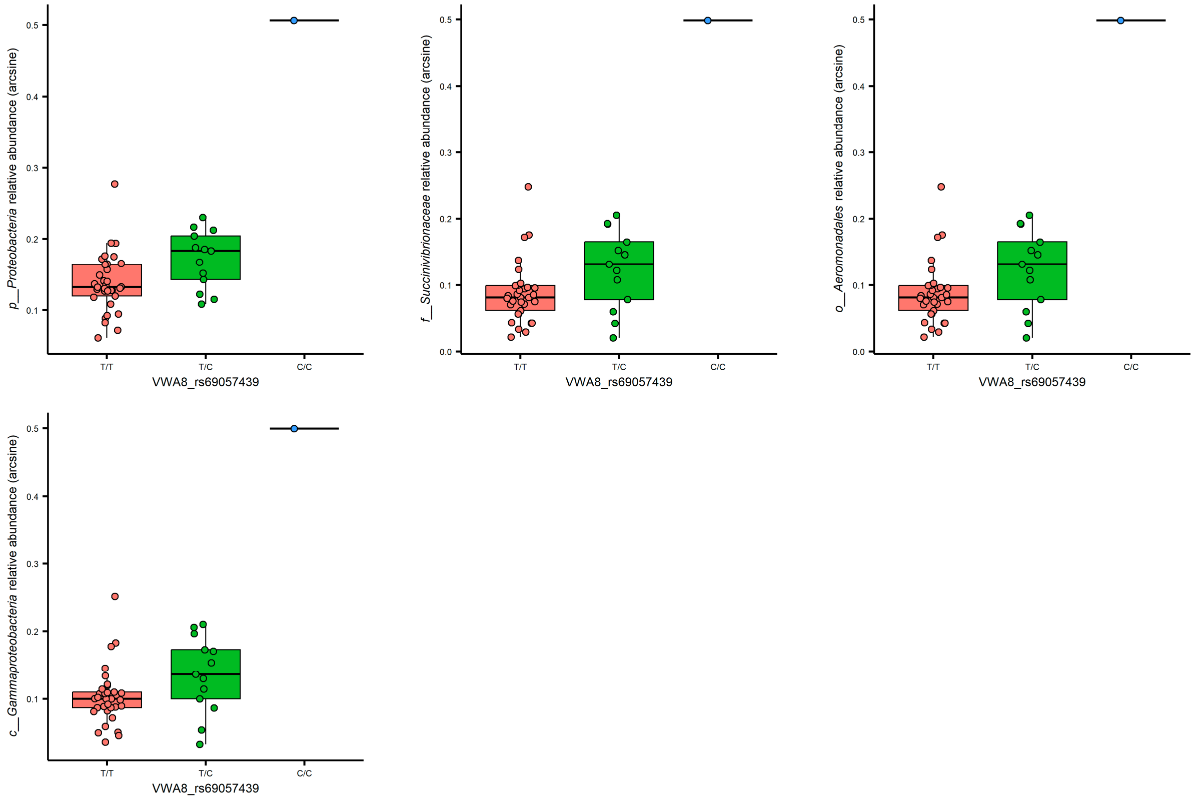

p__Proteobacteria relative abundance (arcsine)
f__Succinivibrionaceae relative abundance (arcsine)
o__Aeromonadales relative abundance (arcsine)
c__Gammaproteobacteria relative abundance (arcsine)
0.0
0.1
0.2
0.3
0.4
0.5
T/T
T/C
C/C
VWA8_rs69057439
